# Imageless robotic handpiece-assisted total knee arthroplasty: a learning curve analysis of surgical time and alignment accuracy

**DOI:** 10.1007/s00402-021-04036-2

**Published:** 2021-07-14

**Authors:** Peter Savov, Lars-Rene Tuecking, Henning Windhagen, Jonathan Ehmig, Max Ettinger

**Affiliations:** Department of Orthopedic Surgery, Hannover Medical School, Anna-von-Borries-Strasse 1-7, 30625 Hannover, Germany

**Keywords:** Computer-assisted surgery (CAS), Total knee arthroplasty (TKA), Learning curve, Robotic handpiece, Kinematic alignment

## Abstract

**Introduction:**

Robotic-assisted surgery techniques are increasing in total knee arthroplasty (TKA). One crucial point is the prolonged time of surgery. The primary objective of this study was to determine the learning curve necessary to minimize the time of surgery. The secondary objective was to evaluate the accuracy of the implant alignment when using an imageless robotic system for TKA.

**Materials and methods:**

In a case–control study, the first 70 consecutive robotic-assisted TKA procedures performed by a single senior surgeon were analyzed with regard to surgery time and implant alignment by comparing the intraoperative plan with the postoperative alignment. The evaluation of the learning curve with respect to surgery time was conducted using cumulative summation (CUSUM) analysis. The joint line height was measured with a new technique. Surgery time and joint line reconstruction were compared to 70 consecutive conventional TKA procedures.

**Results:**

The learning curve for robotic TKA was completed after 11 cases. The learning curve did not influence the accuracy of joint line obliquity, joint line height, or limb alignment. The intraoperative plan designed for the robotic system was precisely implemented. The mean skin-to-skin time in the robotic group after the learning curve was completed did not differ from that in the manual group. A significant positive correlation was observed between the preoperative hip–knee–ankle angle and the postoperative distalization of the joint line in the robotic-assisted TKA group.

**Conclusion:**

After completing the initial learning curve of 11 cases, the surgery time required to perform imageless robotic handpiece-assisted TKA was similar to that for the conventional technique. However, no learning curve was observed for the implant positioning when using the imageless robotic system. The implementation of the intraoperative plan was accurate up to < 2°. The precision of the system allows the implementation of different joint balancing approaches between valgus and varus morphotypes.

## Introduction

Total knee arthroplasty (TKA) is an established treatment for severe osteoarthritis (OA) of the knee joint. In the past, good and very good results were observed following TKA [1–3]. However, approximately 20% of patients remain dissatisfied after TKA [4], typically due to poor alignment, the inaccuracy of implant positioning, changes in the joint line (JL), or soft tissue management during surgery [5–8]. In recent years, several computer-assisted surgery (CAS) techniques, such as robotic-assisted TKA, have been introduced. Several studies have evaluated the potential advantages of implant positioning and soft tissue management of using robotic-assisted TKA versus conventional manual techniques. Robotic-assisted TKA has been associated with a significant reduction in positioning outliers, the more frequent restoration of the natural JL, the successful achievement of target alignment, and reductions in iatrogenic soft tissue injury [7, 9–15].

Modern robotic-assisted TKA systems are either ‘image-based’ or ‘imageless’. Soft tissue properties are collected based on stress range of motion evaluations. The static bony anatomy combined with soft tissue information enables the surgeon to adjust the implant position virtually while obtaining real-time simulations of the effects of changes to alignments and gap balances. To implement the plan, semi-autonomous burrs and saws are used to perform the bony cuts [16, 17].

However, cost efficiency is a potential drawback of robotic-assisted TKA. The potential of prolonged surgical duration after completing the learning curve [13] remains another major concern. In the literature, data concerning the learning curve and the surgical time required to perform robotic-assisted TKA are rare. One study reported short learning curves with respect to the necessary surgical time and the implant positioning when using image-based robotic-assisted TKA [7]. However, no data are yet available reporting on the learning curves for either time or alignment when using imageless robotic-assisted TKA. Further, no information is available regarding the influence of robotic-assisted surgery on shifts to the JL or the influence of the preoperative morphotype on surgical success. Thus, the primary aim of this study was to calculate the initial learning curve necessary for robotic-assisted TKA using an imageless robotic system. The secondary aim was to evaluate the accuracy of the robotic system, the postoperative alignment, and shifts to the JL. Our hypothesis was that after the initial learning curve was complete, that the robotic-assisted surgery time would be comparable to that required for conventional manual surgical techniques and that no learning curve will exist for implant positioning.

## Patients and methods

### Patients

**Fig. 1:**
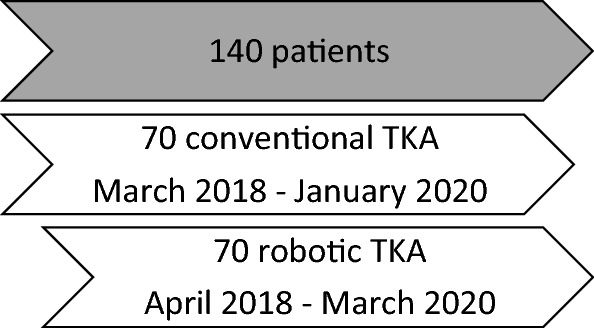
Timeline for the inclusion of all study patients

Between March 2018 and March 2020, the surgical data for 140 consecutive patients who underwent primary TKA (Journey 2 BCS, Smith and Nephew, Memphis, USA) were collected, including 70 consecutive patients through March 2020 who underwent TKA using the conventional technique and the first 70 consecutive patients who underwent robotic-assisted TKA using an imageless robotic NAVIO^®^ Surgical System (Smith and Nephew) performed by a surgeon who was new to the system (Fig. 1). A senior surgeon (M.E.) performed all of the surgeries analyzed for this study. The patients were not randomized, and the surgical technique and whether they received robotic-assisted TKA or conventional TKA were discussed with each patient prior to surgery. No exclusion criteria were applied. All patients had multi-compartmental OA or mono-compartmental femorotibial OA with an insufficient anterior cruciate ligament. The posterior cruciate ligament (PCL) was sacrificed in all cases. This study was approved by the local Ethics Committee (8403_BO_S_2019).

### Parameters

**Fig. 2:**
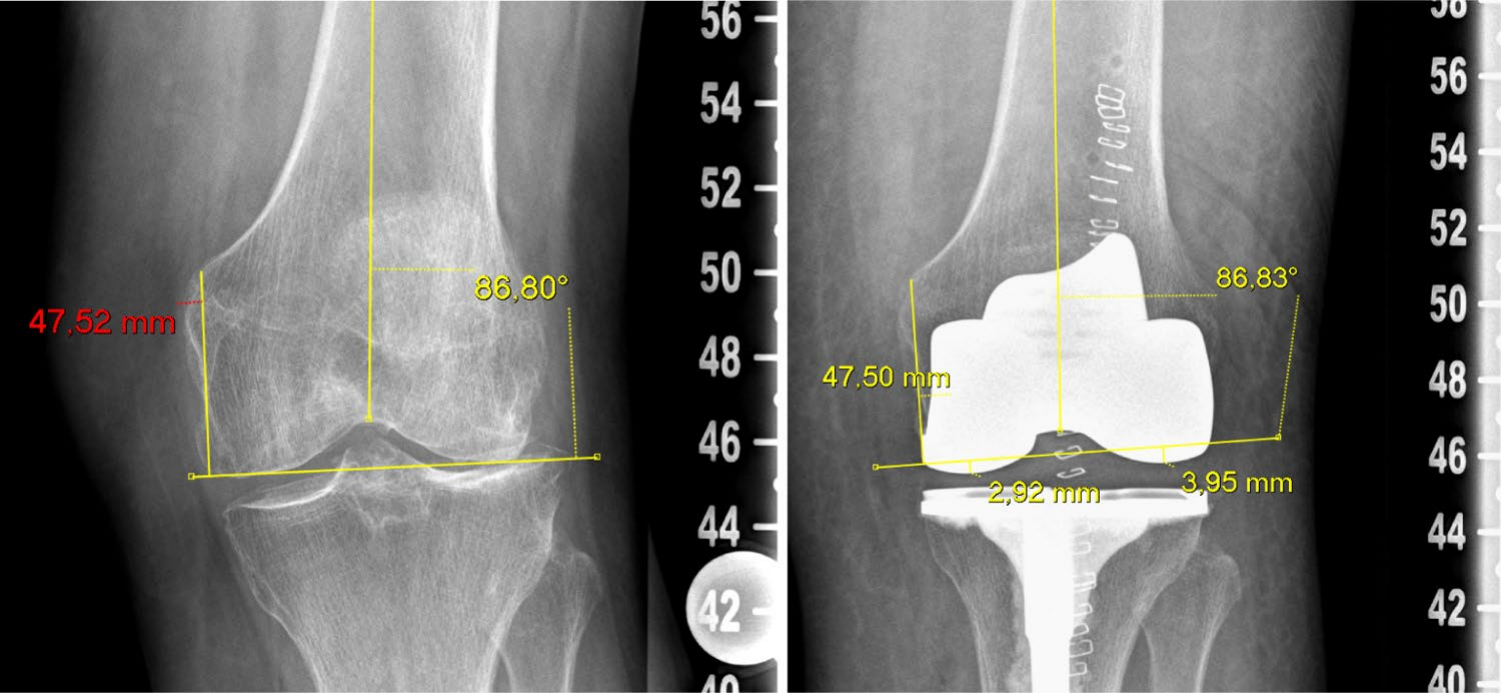
The preoperative joint line obliquity was defined by the LDFA (86.80°). The joint line height was defined as the perpendicular distance to the adductor tubercle of the distal femur, in this case, 47.52 mm. To measure the postoperative shift, we replaced the preoperative joint line (86.83°) in the postoperative radiograph using the same perpendicular distance (47.50 mm) to the adductor tubercle. The shift of the postoperative joint line was defined as the perpendicular distance between the most distal point of the medial (2.92 mm) and lateral (3.95 mm) femoral implant to the preoperative joint line. Distalization was defined as a positive value, and proximalization was defined as a negative value. These values were adjusted to accommodate the natural thickness and elasticity of the femoral cartilage, which is typically defined as 2.00 mm. The adjusted values for the medial and lateral compartments were 0.92 mm and 1.95 mm, respectively. LDFA: lateral distal femur angle

The length of the learning curve with regard to the surgery time was calculated based on the time from skin incision to suture. The accuracy of the intraoperative planned alignment compared with the measured postoperative alignment was calculated for robotic-assisted TKA. The pre- and postoperative alignments for conventional TKA were also documented. The following alignment parameters were analyzed on long-leg radiographs and true lateral radiographs, as described by Paley et al. [18]: hip–knee–ankle angle (HKA), lateral distal femur angle (LDFA), medial proximal tibia angle (MPTA), and posterior slope of the tibia. The HKA is defined as the angle between the mechanical axis of the femur and tibia. The LDFA is defined as the angle between the mechanical axis of the femur and the tangential line of the distal femur. The MPTA is defined as the angle between the mechanical axis of the tibia and the tangential line of the proximal tibia. The posterior tibial slope is defined as the angle between the proximal anatomical axis of the tibia and the tangential line of the medial tibia plateau, which was subtracted from 90° [19]. The JL heights on the medial and lateral sides were measured using a modified measurement technique [20]. Briefly, the preoperative JL obliquity was defined by the LDFA. The JL height was defined as the perpendicular distance to the adductor tubercle of the distal femur. To measure the postoperative JL shift, we replaced the preoperative JL in the postoperative radiographs based on the perpendicular distance to the adductor tubercle. The postoperative JL shift was defined as the perpendicular distance between the most distal point of the medial and lateral femoral implants and the preoperative JL. Distalization was defined as a positive value for the JL shift, whereas proximalization was defined as a negative value (Fig. 2). These values were adjusted according to the natural thickness and elasticity of the femoral cartilage, which is usually defined as 2 mm. All radiographs were calibrated. We used Carestream PACS (Carestream Health, Inc.) to perform all measurements. All parameters were measured once independently by two investigators (P.S. and J.E.), and the overall interrater reliability (Cohen’s kappa) was calculated.

### Surgical technique

Neutral or varus knees were operated using a restricted kinematic alignment (KA) approach [5]. For valgus knees, a mechanical alignment approach was performed. The conventional manual technique was performed using standard instrumentation, a standard medial parapatellar approach, and an extramedullary reference guide for the tibial cut. The PCL was removed in all patients. In cases using the KA technique, the aim was to restore the natural obliquity of the JL and the rotation of the femur with respect to the posterior condylar line. The limit for the tibia vara was set to 87° (MPTA) with respect to the mechanical axis of the tibia. No soft tissue releases were conducted to balance the knee. In valgus cases, the JL (LDFA) was set to 87° with respect to the mechanical axis of the femur (anatomic alignment). The polyethylene onlay was asymmetric, with a varus oblique JL of approximately 3°. The tibia cut was set to 90°. The rotation of the femoral component was set to 0°. All valgus cases were manually redressable. The lateral release of the popliteus tendon (POP), lateral collateral ligament (LCL), or the iliotibial band (ITB) was performed to balance the knee, as necessary.

**Fig. 3:**
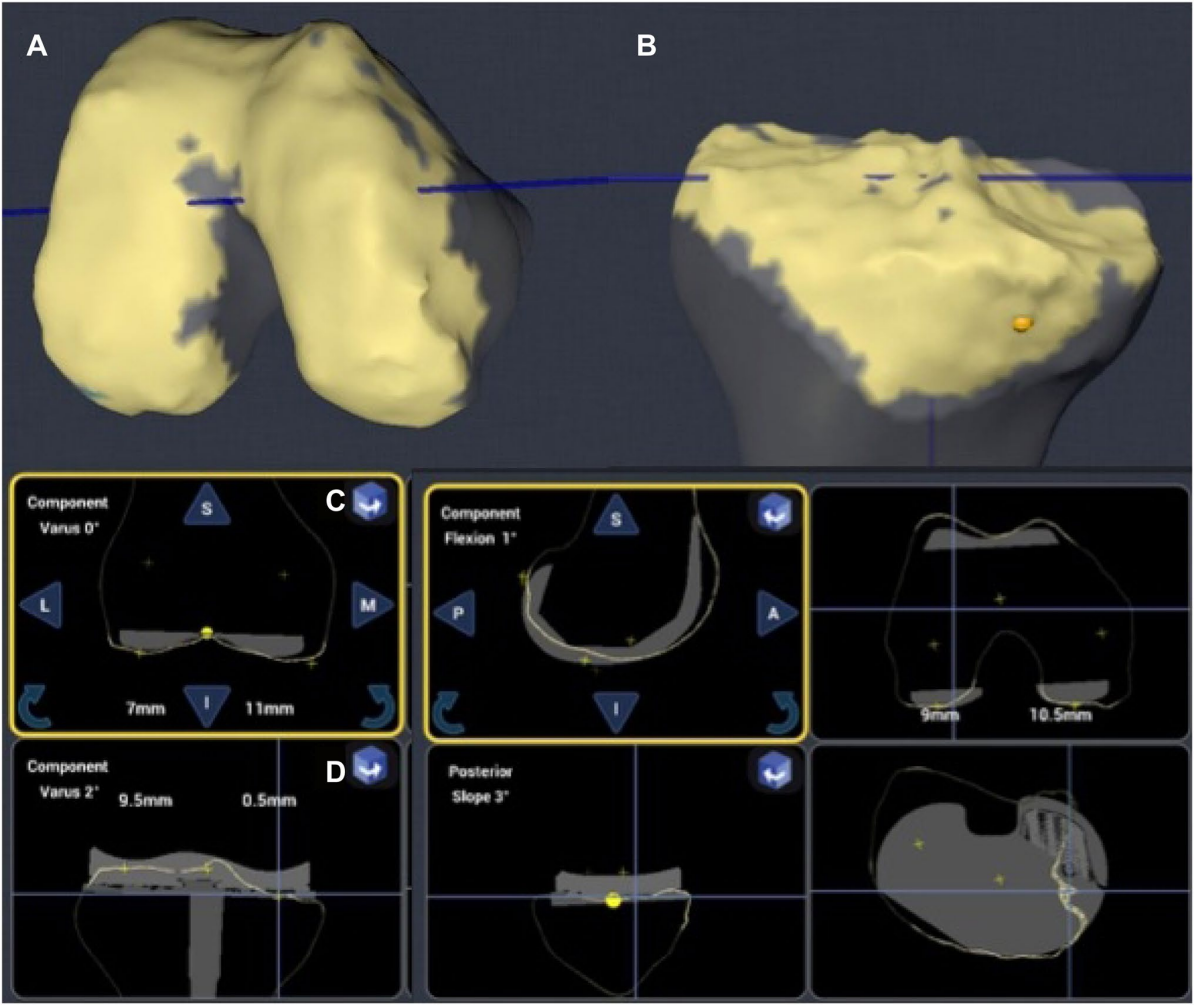
To generate the 3D bone model of the knee joint, the anatomy of the femur and tibia must be painted with the pointer probe (**a**, **b**). Afterward, the position of the prosthesis is planned in all three plains for the femur (**c**) and tibia (**d**), with respect to the extension and flexion gaps throughout the entire range of motion

The robotic-assisted TKA technique began with the setup of the robot, foot-pedals, camera, and handpiece. Procedure selection, handpiece calibration, and burr check-up were performed before performing the skin incision. The time of surgery starts with the initial skin incision, which is performed to place pins in the tibia and femur for the camera trackers. The tension of the collateral ligaments is measured using both valgus and varus stress ranges of motion. To generate a three-dimensional (3D) bone model of the knee joint, the anatomy of the femur and tibia must be painted with the pointer probe. Afterward, the position of the prosthesis is planned with respect to the extension and flexion gaps through the entire range of motion (Fig. 3). Using the handpiece with the semi-autonomous burr, the selected virtual plan for the positioning of the prosthesis is implemented during surgery. The system was used to control all planned cuts. With the trial components, the surgeon was able to check the gaps and the HKA.

### Statistical analysis

To calculate the learning curve required to reach a stable surgery time while using the robotic-assisted procedure, cumulative summation (CUSUM) analysis was used [21]. Using the CUSUM analysis, data could be presented as a running total of the deviations from a target value. Briefly, the deviation of the single surgery time from the overall average time is summed up. The average time was calculated from the surgery time of all included robotic-assisted procedures. CUSUM analysis allows for the visualization of trends or turning points in the investigated performance of the surgeon.

After the learning curve for robotic-assisted TKA was complete, the values for surgery times were compared with the respective values for the conventional manual group. The postoperative alignment parameters were compared with the intraoperative planned values for all robotic-assisted procedures. A subgroup analysis was performed according to the preoperative morphotypes: varus, valgus, and post-traumatic OA. Pearson’s correlation analysis was performed between the preoperative HKA and the postoperative JL shift for the medial and lateral sides in both the conventional and robotic-assisted groups. No overall target alignment was established for the conventional TKA group; therefore, the postoperative alignment of the conventional group was not compared with the robotic-assisted group. The inter-observer coefficient was calculated for all radiographic measurements.

To determine the precision of the robotic system, the absolute deviation from the intraoperative plan and the standard error of the estimate (Sy.x) value was calculated. This value is similar to the root-mean-square error (RSME) and can be determined regardless of whether the data fit the linear regression of the postoperative radiograph and intraoperative plan by calculating the standard deviation of the residuals. To evaluate the mean differences, the Student’s *t* test was used. The level of significance was set to 0.05. For subgroup analyses, the level of significance was adjusted using the Bonferroni correction. To test the equivalence of the surgery time, the 90% confidence interval was calculated. If the full range of the confidence interval lies within the determined margin, we can conclude with 95% confidence that the two treatments are equivalent. To achieve adequate power, we relied on previous studies that investigated surgery times between conventional and CAS techniques [7, 22, 23]. The clinically relevant difference in surgery times was set to 5 min, with a standard deviation of 10 min. The mean surgery time required for a robotic-assisted TKA was defined as 69.4 min, based on Kayani et al. [7]. To detect a difference of 5 min using a two-tailed, two-sample, Student’s *t* test with a power of 80% at a significance level of 0.05, this study required a sample size of 128 patients. The sample size was set to 140 patients. All statistical analyses were performed using GraphPad Prism Version 7 (GraphPad Inc., San Diego, CA, USA).

## Results

No significant differences in the demographics were observed between the robotic-assisted TKA and the conventional TKA groups (Table 1).

**Table 1:** Demographics, surgery times after completing the learning curve, and alignment values for the robotic-assisted TKA and conventional TKA groups

	Robotic-assisted TKA group	Conventional TKA group	*P* value
Age	64.4	65.9	0.832
Sex			
Male	22	20	
Female	48	50	
Surgery time (min)	*n* = 59	*n* = 70	
Mean (SD)	69 (± 12)	67 (± 18)	0.491
Min	47	46	
Max	112	116	
BMI	28.8	29.6	0.645
OA pathogenesis			0.001*
Varus (n)	22	46	
Valgus (n)	33	24	
Post-traumatic (n)	15	0	
HKA in °, mean (SD)			
Preoperative	180 (± 10)	177 (± 8)	0.03*
Postoperative	178 (± 3.2)	179 (± 2.9)	0.415
LDFA in °, mean (SD)			
Preoperative	87 (± 3)	87 (± 3)	0.228
Postoperative	87 (± 2.5)	87 (± 2.3)	0.803
MPTA in °, mean (SD)			
Preoperative	88 (± 3.8)	88 (± 3.5)	0.324
Postoperative	89 (± 1.6)	89 (± 2.2)	0.044
Slope in °, mean (SD)			
Preoperative	4.1 (± 2.1)	4.9 (± 3.4)	0.083
Postoperative	2.4 (± 1.8)	7 (± 3)	0.001*

*TKA* total knee arthroplasty, *BMI* body mass index, *OA* osteoarthritis, *HKA* hip knee ankle angle, *LDFA* lateral distal femur angle, *MPTA* medial proximal tibia angle

### Surgery time

**Fig. 4:**
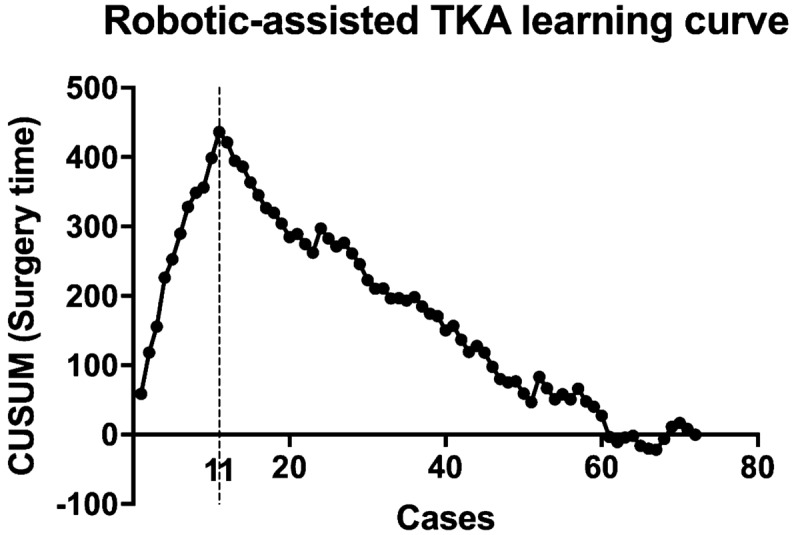
Using the CUSUM analysis, data could be displayed as a running total of deviations from a target value (vertical axis). The CUSUM analysis allows for the visualization of trends or turning points in the investigated performance of the surgeon. The reference values for the surgery time were defined as the average surgery time in minutes for the respective robotic-assisted procedures. The learning curve was completed after 11 cases. *CUSUM* cumulative summation

The CUSUM curve generated for the surgical learning curve is shown in Fig. 4. The turning point was reached after 11 cases. No significant differences were observed for the surgery times between the robotic-assisted TKA and conventional TKA groups after the initial learning curve was completed (robotic-assisted TKA: 69 min vs. conventional TKA: 67 min, P = 0.491). The 90% confidence interval for the mean difference ranges from − 7.9 to + 3.2 min. For testing equivalence, the defined margin of 5 min is within the confidence interval; therefore, equivalence could not be determined (Table 1).

### Alignment

The absolute difference between the postoperative alignment and the intraoperative plan of the robotic-assisted TKA group differed significantly. The MPTA was accurate to 1.0°, the LDFA was accurate to 1.6°, and the HKA was accurate to 2° (Table 2). Post-traumatic OA is a risk factor for outliers with a greater than 3° difference between the intraoperative plan for the LDFA and the HKA and the postoperative outcome for robotic-assisted TKA. Among non-traumatic patients, 5.5% were outliers for the HKA in the robotic-assisted TKA group (Table 3). No learning curve was observed for implant positioning in the robotic-assisted TKA group (Table 4).

**Table 2:** The alignment values of the preoperative and postoperative radiographs and the intraoperative plan of the robotic system for all robotic-assisted TKA patients

Robotic-assisted TKA group (n = 70)	Pre	Intra	Post	Abs. delta	*P* value
HKA (°)	180 (± 10)	177 (± 2.7)	178 (± 3.2)	2.0 (± 1.2)	< 0.001*
MPTA (°)	88 (± 3.8)	88 (± 1.1)	89 (± 1.6)	1.0 (± 0.8)	0.002*
LDFA (°)	87 (± 3)	88 (± 2.1)	87 (± 2.5)	1.6 (± 1.3)	0.014
Slope (°)	4.1 (± 2.1)	2.9 (± 0.7)	2.4 (± 1.8)	1.4 (± 1.3)	0.038

The absolute difference and the *P* value for the comparison of the mean differences between the postoperative X-ray and the intraoperative data were calculated. The *P* value was adjusted to 0.0125, according to Bonferroni. All values are presented as the mean (standard deviation)

*TKA* total knee arthroplasty, *HKA* hip knee ankle angle, *MPTA* medial proximal tibia angle, *LDFA* lateral distal femur angle

**Table 3:** The outliers (± 3° from the planned alignment) in the robotic-assisted TKA group

	All patients	Non-traumatic	Traumatic	*P* value	Odds ratio	95% CI
HKA	8 (11.4%)	3 (5.5%)	5 (33.3%)	0.002*	8.66	1.72–35.28*
MPTA	2 (2.8%)	1 (1.8%)	1 (6.6%)	0.385	3.85	0.19–74.33
LDFA	8 (11.4%)	2 (3.6%)	6 (40%)	< 0.001*	17.67	3.33–90.4*
Slope	2 (2.8%)	1 (1.8%)	1 (6.6%)	0.385	3.85	0.19–74.33

All values are presented as the number and percent of the respective cohorts. The odds ratios of alignment outliers in the robotic-assisted TKA group due to post-traumatic arthrosis were calculated. The *P* value was adjusted to 0.0125, according to Bonferroni

*HKA* hip knee ankle angle, *MPTA* medial proximal tibia angle, *LDFA* lateral distal femur angle

**Table 4:** The Sy.x value was calculated for every 10 cases to determine the learning curve for the precision of the robotic system

Sy.x	Cases 1–10	Cases 11–20	Cases 21–30	Cases 31–40	Cases 41–50	Cases 51–60	Cases 61–70
HKA (°)	2.74	1.39	1.91	2.54	1.03	1.28	2.40
MPTA (°)	1.22	0.94	1.31	1.05	1.07	1.65	1.05
LDFA (°)	1.69	1.00	1.70	1.94	1.86	1.88	2.22
Slope (°)	1.73	1.47	1.15	3.00	1.20	1.31	1.43

The Sy.x is interpreted similarly to the root-mean-square error (RSME). No learning curve was observed for any alignment parameters

*HKA* hip knee ankle angle, *MPTA* medial proximal tibia angle, *LDFA* lateral distal femur angle

### Joint line

**Fig. 5:**
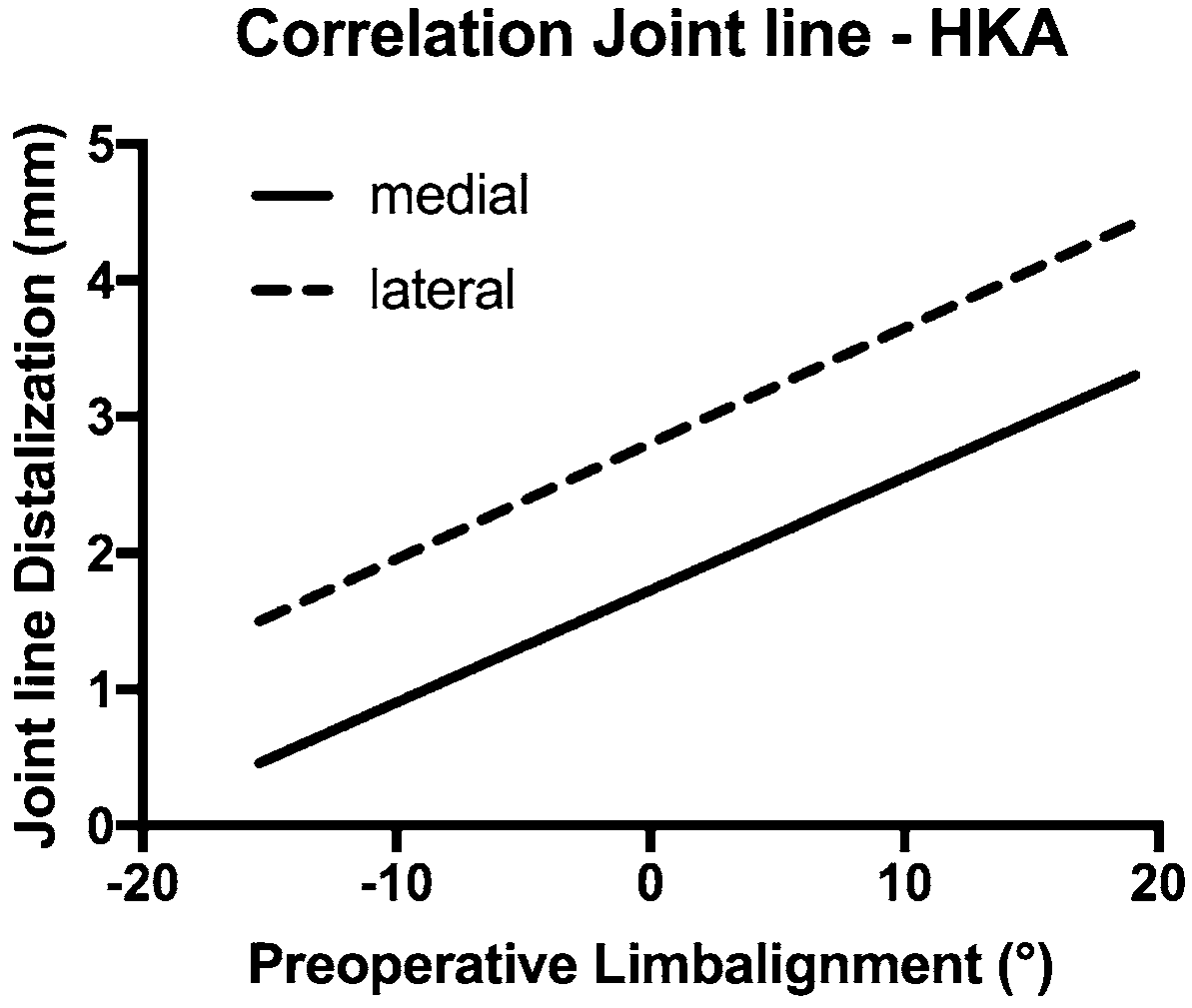
Significant Pearson’s correlation coefficients were calculated between the preoperative overall limb alignment and the postoperative distalization of the medial and lateral parts of the joint line (*P* = 0.001 and *P* = 0.007, respectively). The Pearson’s coefficients were *r* = 0.466 for the medial side and *r* = 0.396 for the lateral side. Negative values on the horizontal axis indicate varus alignment, positive values indicate valgus alignment

The JL in the varus robotic-assisted TKA group was distalized by < 1 mm on the medial side and by < 2 mm on the lateral side. In the varus conventional TKA group, the JL was proximalized on the medial and lateral sides by < 1.5 mm. In the valgus robotic-assisted TKA group, the JL was distalized by 2.57 mm medially and by 3.69 mm laterally. The valgus conventional TKA group showed different results, with the JL shift on the medial and lateral sides equal to almost 0 mm and 1.7 mm, respectively. Significant differences were observed in the JL shift between the robotic-assisted TKA and conventional TKA group in terms of the medial compartment in the valgus morphotypes and the lateral compartment in the varus morphotypes (Table 5). A significant positive correlation was observed between the preoperative morphotype and the post-operative JL shift (Fig. 5). The overall Cohen’s kappa was 0.818 (*z* = 9.89, *P* < 0.001). The Pearson’s correlation coefficients (*r*) between the preoperative HKA and the JL shifts for the medial and lateral sides were 0.466 and 0.396, respectively.

**Table 5:** The shift of the joint line according to groups and subgroups defined by the surgical technique and morphotype

Joint line (mm)	Robotic-assisted TKA group		Conventional TKA group		∂ med	*P* value	∂ lat	*P* value
	Med	Lat	Med	Lat				
Varus	0.9 (± 1.7)	1.9 (± 2.3)	− 0.7 (± 2.4)	− 1.2 (± 2.9)	1.5 (± 0.6)	0.013	3.1 (± 0.7)	< 0.01*
Valgus	2.6 (± 1.6)	3.7 (± 1.8)	− 0.1 ± (2.3)	1.7 (± 3.6)	2.6 (± 0.6)	< 0.01*	1.9 (± 0.8)	0.024

All values are presented as the mean (standard deviation) in millimeters. Significant differences between the surgery techniques regarding the medial compartment in valgus morphotypes and the lateral compartment in varus morphotypes. The *P* value was adjusted to 0.0125, according to Bonferroni

*TKA* total knee arthroplasty

## Discussion

The learning curve for robotic-assisted TKA with an imageless robotic system was completed after 11 cases. The surgery time required for robotic-assisted TKA after completing the initial learning curve was not significantly different from the time required for the manual technique, but no equivalence could be demonstrated. No learning curve was observed for implant positioning with robotic-assisted TKA.

Several key points in the learning curve for robotic-assisted TKA are worthy of discussion. The positioning of the femoral and tibial camera trackers is crucial for the success of the entire procedure. Another contributor to the learning curve when using the imageless robotic system is bone registration. After a few cases, the surgeon is better able to identify the relevant anatomical landmarks necessary for adequate mapping. However, the most challenging component of the learning curve in KA robotic-assisted TKA is the implant positioning with respect to the soft tissues when attempting to reconstruct the individual anatomy of each patient. In our opinion, this step represents the main difference between KA and mechanically aligned robotic-assisted TKA and requires time to learn, especially in the early stages. Kayani et al. reported an initial learning curve of seven cases with an image-based mechanically aligned robotic-assisted TKA [7]. The most time-consuming steps that occur at the beginning of robotic-assisted TKA include system calibration, anatomical registration, and joint balancing. After completing the learning curve, these steps showed the largest improvements in terms of time-saving. These findings are comparable to those reported by Kayani et al. and Sodhi et al. [7, 24]. Thus, the learning curve for TKA with an imageless robotic system is similar to that for an image-based robotic system. Another essential finding of this study was the lack of any significant difference in the overall surgery time between robotic-assisted TKA and the conventional manual technique (69 min [range 47–112 min] vs. 67 min [range 46–116 min], respectively; *P* < 0.05). The time required to perform the robotic-specific surgery steps is similar to the time required for the setup and handling of the alignment guides and cutting blocks used to perform alignment during the conventional method. Previous studies have shown consistent findings [7, 24], although two studies reported significantly longer surgery times for robotic-assisted TKA [25, 26].

When using the imageless robotic system, the implementation of the intraoperative plan was very precise. Given the different alignment philosophies that are applied to the different morphotypes, we compared the postoperative limb alignment with the intraoperative plan rather than with the mechanical axis. The MPTA and LDFA were precisely implemented, with accuracies of 1° and 1.6°, respectively. The standard deviations revealed very small distribution widths of ± 0.8° and ± 1.3°, respectively. The HKA was also precise to within 2° ± 1.2°, regardless of the morphotype. In our opinion, the reduced precision of the HKA compared with those measured for the MPTA and LDFA is likely due to the intraoperative non-weight-bearing measurement method used by the robotic system. These results are consistent with the findings reported in the current literature for robotic-assisted TKA, despite data on imageless robotic systems being limited [7, 25, 27–29]. Two studies have reported on the same imageless robotic system, one of which was a cadaver-based study performed by the manufacturing company [30, 31], and the other performed as a retrospective clinical study [31]. Bollars et al. reported similar accuracy values to ours but with a higher rate of outliers, especially for the femoral component. However, they compared the accuracy to the mechanical axis, and no information was provided regarding the intraoperative plan, preoperative alignment, or morphotype. The target of their study was the mechanical alignment, and outlier rates of 14% for the LDFA and 6% for the HKA were reported [31]. We also observed higher outlier rates for the femoral component and the HKA than for other parameters (Table 3). The majority of our patients with an outlier value had post-traumatic OA. In our study, a significant difference was noted (odds ratio 17.67, 95% confidence interval 3.33–90.4) for a femoral outlier outcome in cases of post-traumatic OA. In our opinion, hidden osteophytes, contracted soft tissue, and poor bone quality play vital roles in increasing the risk of an alignment outlier. We did not compare the accuracy of the robotic-assisted TKA with the conventional technique because no quantifiable alignment target can be defined for the manual KA technique. No learning curve was observed for the implant positioning in the robotic-assisted TKA group. The Sy.x parameters were calculated for every 10 consecutive patients (Table 4), and no significant differences were observed between the Sy.x values of each alignment parameter. These findings are comparable to those reported by Kayani et al. [7]. Intraoperative virtual joint balancing with respect to the individual soft tissue envelope and precise bone resection immediately resulted in the intended outcome for both image-based and imageless robotic-assisted TKA.

An elevation in the JL is negatively correlated with clinical outcomes after TKA [32]. This is the first study to investigate differences in the JL shift of the medial and lateral compartments after robotic-assisted TKA. We showed that the JL was not proximalized during robotic-assisted surgery. Rather, a slight distalization occurred. A significant correlation was observed between the preoperative HKA and the extent of the JL shift (Fig. 4). The mean distalization values for the medial and lateral sides of the JL in the valgus morphotype subgroup were approximately 2.6 ± 1.6 mm and 3.7 ± 1.8 mm, respectively; in the varus morphotype subgroup, the respective values were 0.9 ± 1.7 mm and 1.9 ± 2.3 mm. In our opinion, this interesting deviation is likely due to the soft tissue alterations that occur with a valgus morphotype [33]. With the robotic system, the surgeon has precise control over joint balancing and might use distalization of the femur to tighten the extension gap. Interestingly, the conventional TKA group showed a slight proximalization of the JL, especially in the varus group, and a significantly larger standard deviation from the mean value was observed in the conventional TKA group compared with that in the robotic-assisted TKA group. The differences between the postoperative JL values of the robotic-assisted TKA and conventional TKA groups were partially significant. Therefore, we hypothesized that the proximalization of the JL, which could lead to a negative outcome, could be avoided through the use of the robotic system.

No data are available in the literature that can be used to differentiate the JL distalization of the femoral compartments.

Our study has some limitations. First, the accuracy and JL measurements were performed on standard, weight-bearing, long-leg radiographs, which introduces known inaccuracies compared with computed tomography (CT)-based measurements. The quality of the radiograph is crucial for the success and accuracy of the measurements. However, long-leg radiographs show good validity compared with 3D CT scans [34]. The quality of the JL measurement is based on an intact bone stock on the femoral side. In cases of severe OA with femoral bone loss, measurement errors can occur. Second, the groups were slightly inhomogeneous due to the use of a single senior surgeon, the consecutive series, and the willingness of the patients to be operated using a robotic system. Therefore, significant differences in the preoperative alignments and morphotypes were observed between the groups. However, in our opinion, these differences did not significantly influence the investigation of the primary and secondary objectives. This study was not intended to be performed as a randomized controlled study. Third, we did not examine the clinical results and long-term data regarding implant survivorship and revision rates.

## Conclusion

Once the initial learning curve for robotic-assisted TKA has been completed, the surgery times are similar to those for the conventional manual technique, with no learning curve for implant positioning. The precision of the system might result in different joint balancing between valgus and varus morphotypes. A significant correlation between the preoperative HKA and the postoperative distalization of the JL was observed.
